# Comparative efficacy of traditional conservative treatment and CT-guided local chemotherapy for mild spinal tuberculosis

**DOI:** 10.1186/s12891-022-05545-w

**Published:** 2022-06-18

**Authors:** Yangyang Guo, Meitao Xu, Lei Li, Bin Gu, Zehua Zhang, Wenbo Diao

**Affiliations:** 1 Department of Orthopaedics, Zhoukou Orthopedic Hospital, Eastern Taihao Road, Zhoukou City, Henan Province, China; 2 Department of Orthopaedics, Southwest Hospital, Third Military Medical University, Chongqing, China

**Keywords:** Conservative treatment, Clinical outcome, Spinal tuberculosis, Kyphosis

## Abstract

**Background:**

There are considerable differences in the treatment strategy for spinal tuberculosis, including conservative or surgical procedures. Conservative treatment is always suitable for most patients. This study aimed to compare the clinical efficacy of traditional conservative treatment with CT-guided local chemotherapy strategy of mild spinal tuberculosis.

**Methods:**

This research retrospectively analysed 120 patients with spinal tuberculosis between January 2005 and January 2016 according to the diagnostic criteria of mild spinal tuberculosis. In total, 89 patients underwent traditional conservative treatment, 31 underwent CT-guided local chemotherapy. Clinical outcome, laboratory indexes, and radiological results were analysed to provide a clinical basis for the choice of mild spinal tuberculosis treatment.

**Results:**

All cases achieved a clinical cure with 24 to 50 months followed up. Cobb angle of the two groups spinal tuberculosis segments was 6.25 ± 3.1100B0, 5.69 ± 2.5800B0 before treatment and 12.36 ± 6.3100B0, 14.87 ± 7.2600B0 after treatment, respectively. The VAS scores were significantly decreased post-treatment. At the 1 month follow-up, the VAS scores and erythrocyte sedimentation rate (ESR) were significantly differences between the two groups. The efficacy in the CT-guided local chemotherapy (Group B) was better than the traditional conservative treatment (Group A). But from the 3 months follow-up to the last follow-up, the VAS scores and ESR was no significant differences between the two groups and the average ESR decreased to normal. There was no evident kyphosis, symptoms or neurological deficits at the final follow-up. The paravertebral abscesses had disappeared, with no significant progression of local kyphosis, significant absorption and clear lesion edges, pain relief and normal ESR in the two groups.

**Conclusions:**

For mild spinal tuberculosis, traditional conservative treatment can achieve satisfactory results. The strategy combined with CT-guided local chemotherapy treatment is minimally invasive, beneficial for the drainage of paravertebral abscesses and pain relief.

## Background

Spinal tuberculosis is the most common and serious form of tuberculosis lesion in skeleton and can result in severe complications including kyphosis and paralysis [1, 2]. Although the cure rate of spinal tuberculosis is high with the use of anti-tuberculosis drugs, there is still no consensus on the use of chemotherapy, surgical indications, and surgical methods related to spinal tuberculosis [3, 4]. The blind increase in surgical indications and over-operation have become problems that need to be solved urgently. How to properly select conservative treatment or surgery has gradually attracted the attention of clinicians. Several researchers have attempted to classify spinal tuberculosis in order to standardise the treatment strategy but classification method havebeen widely accepted due to some shortcomings [5, 6].

Through a literature review and in an effort to summarise our relevant clinical experience, we have suggested a standard for the early diagnosis of mild spinal tuberculosis [6]. The patients from this study, which were treated with a traditional conservative approach, were compared to CT-guided local chemotherapy treatment. The efficacy and safety were evaluated to improve the treatment strategy for spinal tuberculosis.

The study was approved by the Institutional Ethics Review Board of the First Affiliated Hospital at Third Military Medical University and the Ethics Committee of the Zhoukou Union Orthopedic Hospital. Written informed consent was obtained from all patients or their guardians and retrospectively registered. The authors declare that this report does not contain any personal information that could lead to the identification of the patient(s) and/or volunteers.

## Methods

Between January 2005 and January 2016, 120 mild spinal tuberculosis cases were included by imaging, pathology, tuberculosis or gene chip by bacterial culture in this research. 89 patients underwent traditional conservative treatment (Group A), another 31 cases were performed CT-guided local chemotherapy (Goup B). The patient's treatment of choice was determined involved a multi-discipline team and participation with the patient and his families also, which was led by spine surgeons, participation with an interventional and diagnostic radiology physician specialist, a pathologist, a physician or infectious diseases specialist. All written informed consents of treatments was obtained from the patient.

The word meaning of mild spinal tuberculosis were as follows: active spinal tuberculosis, mild systemic and local symptoms; single central vertebral or two margin vertebral tuberculosis; spinal appendage tuberculosis without spinal canal involvement; mild bone destruction (< 1/3 vertebral height, no large sequestrum); no retropharyngeal, single vertebral involvement with central lesion or multivertebral involvement with edge type lesion; neurological deficits according with American Spinal Injury Association (ASIA) D or normal; no obvious kyphosis (Cobb angle < 3000B0); and no obvious spinal instability. We chose the mild spinal tuberculosis patients in this study, the inclusion criteria of the traditional conservative treatment (Group A) was the paravertebral abscess less than 50 ml based on imaging, if the paravertebral abscess was > 50 ml, the CT-guided local chemotherapy (Group B) were performed. The exclusion criteria were as follows: severe bone destruction (> 1/3 vertebral height), significant kyphosis (Cobb angle > 3000B0), obvious neurological deficit (worse than ASIA D), large abscesses and accompanying sequestrum requiring debridement, bone grafting and internal fixation, complicated active pulmonary tuberculosis, and poor tolerance or compliance.

The traditional conservative treatment (Group A): the 89 patients without paravertebral abscesses or psoas gravitation abscess were accepted four drug anti-tuberculous chemotherapy, i.e. HREZ (rifampicin, 15 mg/kg, maximum, 600 mg/day; isoniazid, 6 mg/kg, maximum 300 mg/day; ethambutol, 25 mg/kg, maximum 750 mg/day; pyrazinamide, 25 mg/kg, maximum 750 mg/day) for at least 18 months. In order to improve the compliance and convenience of patients outside the hospital, all patients and their families received education and training to make clear the importance of early, regular, appropriate, whole-course, and combined medication, and to supervise the patients’ medication.

The CT-guided local chemotherapy (Group B): the 31 patients appeared obvious paravertebral abscesses or psoas gravitation abscess, in the prone or lateral position, a CT positioning scanner was used to select the best puncture point. The punctures were performed above the transverse process and into the intervertebral space and paraspinal abscess. Under local anaesthesia, puncture was performed with an 18G puncture needle to reach the lesion position. After CT reconfirmation, the guide wire and expansion tube were taken out and the double-lumen tube was placed inside. The patient was observed for 30 min. Vital signs were monitored and antibiotics were used to prevent infection for three days after the operation (1 g rifamycin sodium or 0.6 g isoniazid was added to 500 ml saline for 24 h under continuous infusion). Four drug anti-tuberculous chemotherapy HREZ was performed following the same procedure as that for the group A.

All patients were followed up 1, 3, 6, 9, 12, 18 months after treatment, and then once a year thereafter). Clinical symptoms, signs, and ESR, liver and kidney function were monitored. A visual analogue scale (VAS) was used to assess pain improvement, along with a neurological status test according to the American Spinal Injury Association (ASIA) impairment scale [7]. All patients underwent imaging examination (X-ray, CT, MRI). Radiology (X-ray, CT) was used to evaluate kyphosis. VAS score and kyphosis Cobb angle were measured before and after the treatment.

Cure definition was as follows: follow-up more than 18 months, good body condition, normal temperature, good appetite, normal ESR, no obvious local symptoms, no abscess or sinus, X-ray shows abscess disappearance or calcification, no sequestrum or absorption, and lesion edge clear or joint fusion [8, 9]. All patients received nutritional supporting treatment. Conservative treatment patients were not confined to bed but told to avoid strenuous exercise.

All analyses were performed using the SPSS statistical software package (version 14.0, SPSS Inc, Chicago, IL, USA). The progression of VAS and ESR at the before treatment, various follow-up timepoints at 1, 3, 18 months of treatment and the last follow-up were compared between the two groups using repeated measure analysis. The before treatment and final follow-up kyphotic angles were compared with analysis of variance among groups, and then using the Student–Newman–Keuls test to compare each group. Gender was statistically analysed using the χ^2^ test, and age was statistically analysed using analysis of variance. *P* < 0.05 was considered to indicate a significant difference.

## Results

**Fig. 1:**
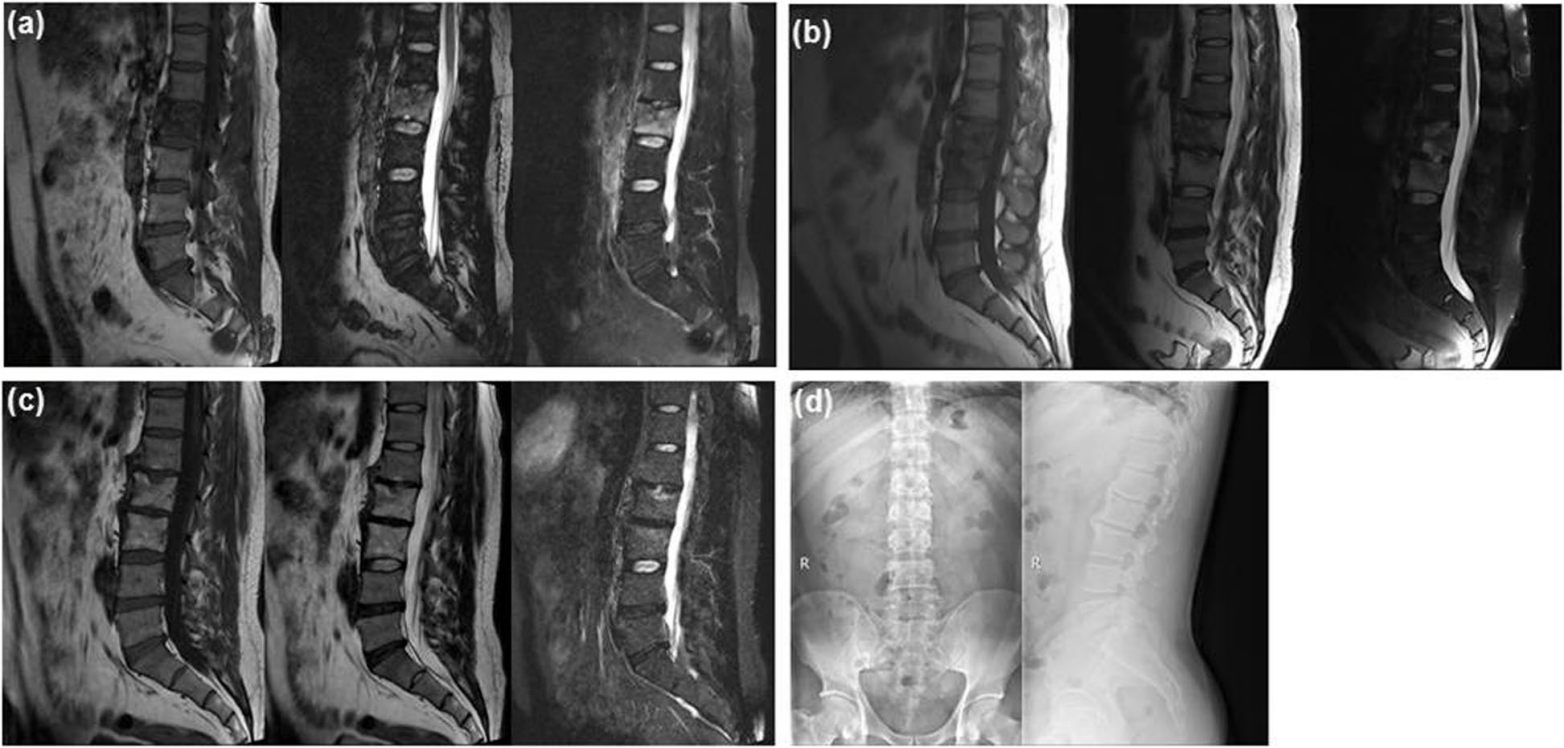
**a** Sagittal magnetic resonance imaging (MRI) showing a 22-year-old male with mild spinal tuberculosis at the L2 vertebral body. The patient was treated with standard first-line antituberculosis drugs for months. **b** At the 6 months follow-up, the sagittal MRI showed significant edema at the L2 and L3 levels, bone destruction exacerbated without obvious abscess, nerve dysfunction, kyphosis and spinal instability. Conservative treatment was continued. **c** Sagittal MRI showing significant absorption and a clear lesion edge at 18 months follow-up. **d** The anteroposterior radiography showing the two years after 18 month of standard chemotherapy treatment. The patient was considered cured and solid fusion appeared at the L2/3 levels, without any complications such as kyphosis or spinal instability.

The general data of patients was described (Table 1). 120 mild spinal tuberculosis patients were included in this research, including 89 males and 31 females with an average age of 28.08 ± 10.78 years (range 15–78 years). All patients were followed up for 24–50 months with an average 33.45 ± 13.07 months (range 24–50 months). In the traditional conservative treatment (Group A), at the one month follow-up, the ESR dropped in 85 patients, and then returned to a normal level at three months, but increased in four lumbar patients, accompanied by obvious pain, aggravated bone destruction, progressive kyphosis, unabsorbed paravertebral abscess. These patients underwent further surgical treatment. The drug sensitivity test indicated they were infected with resistant spinal tuberculosis, so chemotherapy drugs were adjusted, and the patients were declared cured 18 months after surgery. All patients were able to follow the chemotherapy regimen for 18 months and achieved clinical cure (95.51%) (Fig. 1), except the 4 patients infected with resistant spinal tuberculosis had been treated with correction, bone grafting surgery.

**Table 1:** Patient data

Group	Group A (*n* = 89)	Group B (*n* = 31)	*P* value
Gender			0.496^a^
Male	55	17	
Female	34	14	
Age	28.08 ± 10.78	26.87 ± 9.56	0.531^b^
Distribution			_
Cervical	2	0	
Thoracic	21	10	
Thoracolumbar	14	12	
Lumbar	50	9	
Lumbosacral	2	0	

^a^ There was no significant difference in gender between the two groups (Chi-square test, *p* > 0.05)

^b^ There was no significant difference in age between the two groups (Analysis of variance, *p* > 0.05)

**Fig. 2:**
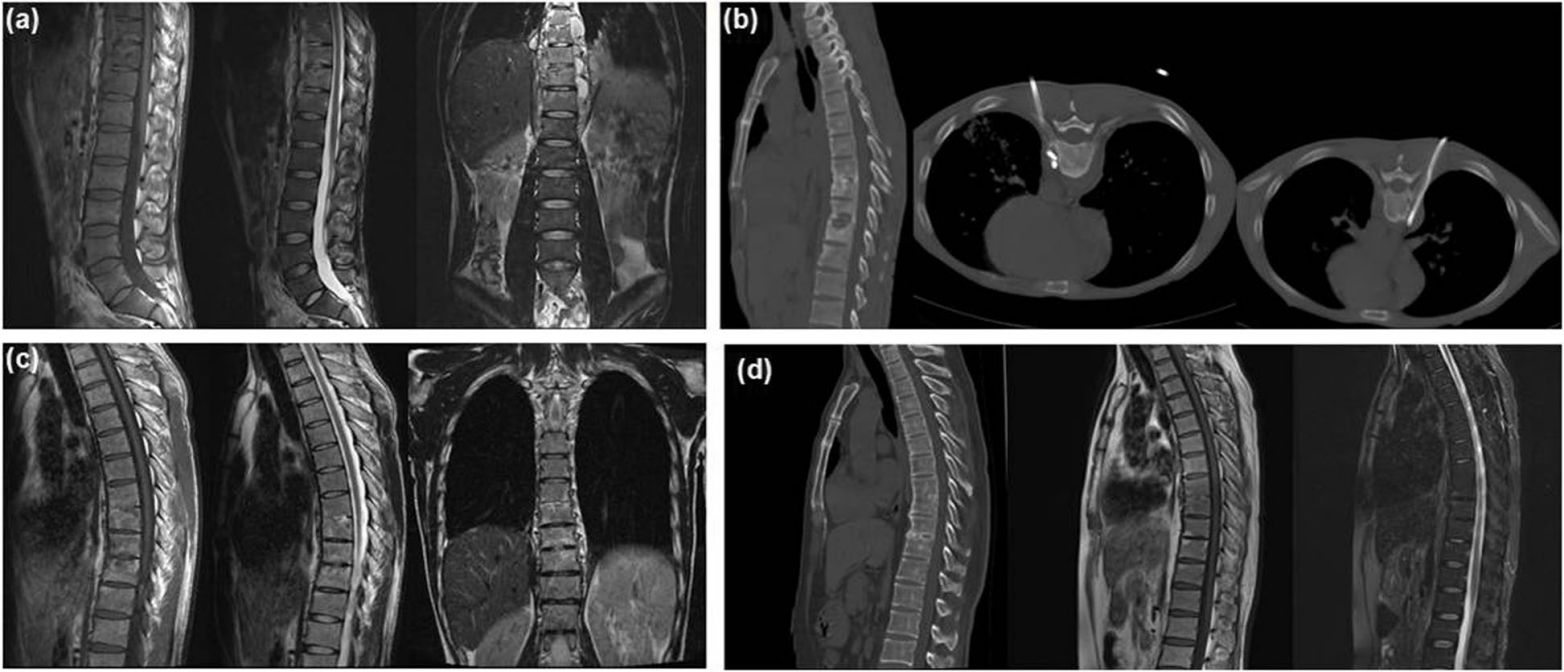
**a** Sagittal and coronal MRI images showing a 31-year-old male with mild spinal tuberculosis at the T8 ~ T11 levels. Tuberculosis accumulated in the vertebral body and intervertebral space with a smaller paravertebral abscess; no distinct kyphosis was observed. **b** Sagittal and axial CT scan showing tuberculosis of the thoracic vertebra. An abscess was treated with CT puncture, followed by two months of drainage.**c** At one year follow-up, sagittal and coronal MRI showed significant bone destruction and intervertebral space reduction, but the tuberculosis abscess has disappeared. **d** Four years after combined treatment with CT guided puncture, catheter drainage and 18 months of standard chemotherapy, sagittal CT and MRI showed a solid fusion at the T8/T9, T10/11 levels and destruction of the thoracic vertebrae. The paravertebral abscesses had disappeared at the T8 ~ T11 levels, with no significant progression of local kyphosis.

In the CT-guided local chemotherapy (Group B), the drainage time was three weeks to three months in patients with catheter drainage, local intensified chemotherapy and with combined drug treatment for 18 months. The extraction standard of the drainage tube was drainage pus less than 5 ml/day, observed continuously for 3 days, ESR < 20 mm/h and abscess disappearance by CT or MRI re-examination. During the treatment period, all patients were treated successfully without cross-infection. At one month follow-up, one patient showed increased ESR, pain and progressive kyphosis, then underwent surgical treatment. The drug sensitivity test indicated it was due to resistant spinal tuberculosis, so the chemotherapy drugs were adjusted and the patient was cured 18 months later. The remaining patients were clinically cured and the abscesses disappeared. MRI showed a vertebral signal similar to the surrounding normal vertebral body (Fig. 2).

In the conservative treatment group (Group A), most patients with out obvious abscess still had pain at the first month follow-up. At the three months follow-up, X-rays showed that the bone was stable in the vertebral destruction area, the marginal bone appeared sclerotic, the pain had disappeared, and MRI showed no obvious abscess. At six months follow-up, 36 patients showed interbody fusion. At nine months follow-up, 67 showed bone fusions. At 18 months follow-up, there were 85 cases of interbody fusion, the disease was cured except the 4 resistant spinal tuberculosis. In the CT-guided puncture/irrigation combined conservative treatment groups (Group B), the abscess disappeared, and the pain symptoms were relieved one month postoperatively except the one resistant spinal tuberculosis. At three to six months postoperatively, the lesion showed marginal sclerosis and intervertebral fusion. Vertebral body fusion was observed at one year follow-up except the one resistant spinal tuberculosis.

The VAS scores of two groups were significantly decreased post-treatment. At the 1 month follow-up, the average VAS scores was 4.75 ± 1.82 in the group A and 3.05 ± 1.37 in the group B, the average ESR was 30.54 ± 9.15 (mm/h) in the group A and 20.20 ± 8.65 in the group B, which were significantly differences between the two groups. The efficacy in the CT-guided local chemotherapy (Group B) was better than the traditional conservative treatment (Group A). But from the 3 months follow-up to the last follow-up, the VAS scores and ESR was no significant differences between the two groups and the average ESR decreased to normal (Tables 2 and 3).

**Table 2:** Comparison of VAS score showing the progression at the various follow-up timepoints of treatment between the two groups

Group	Before treatment	At the 1 month	At the 3 months	At the 18 months	Last follow-up
Group A	5.61 ± 1.63	4.75 ± 1.82	3.05 ± 1.53	2.56 ± 1.44	2.06 ± 1.07
Group B	5.23 ± 1.23	3.05 ± 1.37	2.86 ± 1.67	2.19 ± 1.34	1.81 ± 1.01
*P* value	0.075^a^	0.045^b^	0.231^c^	0.416^d^	0.384^e^

^a^ There was no significant difference in VAS score before treatment between the two groups (Repeated measure analysis, *p* > 0.05)

^b^ There was significant difference in VAS score at the 1 month of treatment between the two groups (Repeated measure analysis, *p* < 0.05)

^c^ There was no significant difference in VAS score at the 3 months of treatment between the two groups (Repeated measure analysis, *p* > 0.05)

^d^ There was no significant difference in VAS score at the 18 months of treatment between the two groups (Repeated measure analysis, *p* > 0.05)

^e^ There was no significant difference in VAS score at the last follow-up between the two groups (Repeated measure analysis, *p* > 0.05)

**Table 3:** Comparison of ESR (mm/h) showing the progression at the various follow-up timepoints of treatment between the two groups

Group	Before treatment	At the 1 month	At the 3 months	At the 18 months	Last follow-up
Group A	71.52 ± 14.85	30.54 ± 9.15	15.37 ± 3.17	10.56 ± 2.35	9.32 ± 2.07
Group B	73.23 ± 18.79	20.20 ± 8.65	10.05 ± 2.44	9.87 ± 2.61	8.81 ± 1.75
*P* value	0.954^a^	0.036^b^	0.738^c^	0.655^d^	0.712^e^

^a^ There was no significant difference in ESR before treatment between the two groups (Repeated measure analysis, *p* > 0.05)

^b^ There was significant difference in ESR at the 1 month of treatment between the two groups (Repeated measure analysis, *p* < 0.05)

^c^ There was no significant difference in ESR at the 3 months of treatment between the two groups (Repeated measure analysis, *p* > 0.05)

^d^ There was no significant difference in ESR at the 18 months of treatment between the two groups (Repeated measure analysis, *p* > 0.05)

^e^ There was no significant difference in ESR at the last follow-up between the two groups (Repeated measure analysis, *p* > 0.05)

At the last follow-up, the average Cobb angle was 12.36 ± 6.3100B0 in conservative treatment (Group A), 14.87 ± 7.2600B0 in CT-guided local chemotherapy (Group B), with no significant differences. Compared with the pre-treatment Cobb angle, mild residual kyphosis was observed in the two groups but no significant differences between the two groups (Table 4). No obvious symptoms of neurological deficits were observed, and all patients returned to normal daily activities. Except the patients infected with resistant spinal tuberculosis had been treated with correction, bone grafting surgery, the other patients were cured from the18 months follow-up to the last follow-up. The paravertebral abscesses had disappeared, with no significant progression of local kyphosis, significant absorption and clear lesion edges, pain relief and normal ESR in the two groups.

**Table 4:** Comparison of Cobb angle before and after treatment between the two groups

Group	Before treatment	Last follow-up
Group A	6.25 ± 3.11°	12.36 ± 6.31°
Group B	5.69 ± 2.58°	14.87 ± 7.26°
*P* value	0.061^a^	0.093^b^

^a^ There was no significant difference in Cobb angle before treatment between the two groups (Analysis of variance, *p* > 0.05)

^b^ There was no significant difference in Cobb angle at last follow-up between the two groups (Analysis of variance, *p* > 0.05)

## Discussion

The best basic treatment of spinal tuberculosis diseases is chemotherapy, rest, and immobilisation. It was advocated chemotherapy alone at the beginning until Hodgson advocated debridement and bone grafting fusion surgery [10]. Surgery should be considered in cases of unstable spine to prevent kyphosis, progressive symptoms of spinal cord nerve damage to avoid paralysis. The final neurological improvement is significantly affected by patients suffering from tuberculosis of spine, vertebral involvement, AIS grade, bladder and bowel involvement and its duration [11]. Complete debridement and decompression can reduce the paralysis, re-establish spinal stability and correct the deformity, as well as shorten the treatment cycle, reduce adverse drug reactions, and improve quality of life [12, 13].

Surgical strategy has significant advantages in terms of preventing kyphosis progression and neurological deficits, but with many disadvantages such as considerable trauma, risk to important organs, blood vessels and nerves, postoperative complications, economic burden and so on. Selecting the appropriate treatment programme for spinal tuberculosis is challenging for surgeons [14, 15]. The choice of surgery or drug therapy has yet been reached an agreement. Many studies have reported satisfactory results for the treatment of spinal tuberculosis with simple chemotherapy, and about 80% of patients achieved spontaneous fusion. Chemotherapy became one essential foundation for the treatment of spinal tuberculosis [16, 17]. The antitubercular drugs are the key stones in the management of tuberculosis of spine, similar as soft tissue tuberculosis management, limited role of surgery [18]. For patients without serious complications, drug chemotherapy can achieve satisfying short and long-term effects. Moreover, the conservative treatment is suitable for most patients, as an early incarnation of the concept of individualised treatment. Bhojraj et al. [19] reported more than 98% spinal tuberculosis patients can be cured by simple chemotherapy and avoided surgery. No significant difference in functional outcome was found between conservative management and surgery for cases with uncomplicated spinal tuberculosis [20].

The indication for the conservative treatment of spinal tuberculosis has always been controversial. Conservative treatment should be suitable for patients without obvious kyphosis, spinal instability, and progressive dysfunction of the spinal cord. In our past research, we characterised a subtype of spinal TB called ‘mild spinal TB’ in great details and selected the study population accordingly to improve the clinical classification and treatment of spinal tuberculosis [6]. In a prospective study, patients without neurological deficits and significant kyphosis were treated effectively with anti-tuberculosis drugs [21]. The results showed that 42 patients (95.4%) were clinically cured without any significant kyphosis after only chemotherapy. In our study, the average number of spinal tuberculosis levels involved was 2.5 (range from one to five levels). Single vertebral involvement with a central lesion or multivertebral involvement with edge type lesions are indications for conservative treatment. We found that patients who underwent CT-guided local chemotherapy for spinal tuberculosis involving with paravertebral abscess or psoas gravitation abscess, also achieved satisfactory results without obvious kyphosis and no spinal instability.

Although mild kyphosis was observed, there were no obvious symptoms in the two groups. With progress in treatment concepts, mild spinal tuberculosis is no longer treated surgically in recent years, but rather most patients are treated by simple chemotherapy and achieve satisfactory results. The existence of tuberculosis cold abscess always causes toxemia, abnormal inflammatory laboratory indicators, increased consumption, and pain. It's theoretically possible that traditional drug chemotherapy is not easy to obtain early control of abscess, the CT puncture treatment can play the purpose of rapid recovery of eliminate pus and pain relief. In this study, at the 1 month follow-up, the VAS scores and ESR were significantly differences between the two groups. The efficacy in the CT-guided local chemotherapy(Group B)was better than the traditional conservative treatment (Group A). But from the 3 months follow-up to the last follow-up, the VAS scores and ESR was no significant differences between the two groups and the average ESR decreased to normal, which milght benefits from basic chemotherapy. It is very likely that patients in the traditional chemotherapy group had less or small abscess, and the chemotherapy drugs could be effectively controlled TB infection in the early stage. Minimally invasive surgery involved CT-guided percutaneous catheter drainage and percutaneous catheter infusion chemotherapy carries advantages in terms of less invasiveness, precise drainage, and enhanced local drug concentration. The use of CT-guided percutaneous may be recommended in addition to conservative chemotherapy and open debridement and instrumental fixation for patients with paravertebral or psoas abscesses and spinal tuberculosis [22]. There was no obvious difference between traditional conservative treatment with drug anti-tuberculous chemotherapy and CT-guided local chemotherapy to delay kyphosis, spinal instability, and neurological deficits in mild spinal tuberculosis by comparison.

With the development of the economy, the health consciousness of patients has gradually improved, and various diagnostic techniques have been applied to the clinic. These affordable and simple actions and district levels could facilitate earlier diagnosis [23]. Diagnosis of spinal tuberculosis (TB) in the early inflammatory stage is essential to prevent the development of spinal deformity and neurological deficit [24]. Due to this, the early diagnosis rate of spinal tuberculosis has been significantly enhanced. Before the symptoms of kyphosis, gravitation abscess, neurological deficits, and spinal instability occur, patients can be diagnosed and treated early. The CT puncture treatment technique has some advantagesis of less traumatic or risky, beneficial for obtaining specimens, detecting drug resistance and acomplishing early individualised treatment.

One persistent controversy in the treatment of spinal tuberculosis is the absence of a general classification system to guide clinical protocols. In order to standardise treatment strategies, a few researchers have attempted to classify spinal tuberculosis, but this has not been widely accepted because of some shortcomings. The classic pathological types (edge type, central type, sub-ligament type, accessory type) are too simple, making it difficult to guide clinical surgical decisions and prognosis. In 2001, Mehta et al. [25] divided thoracic vertebrae into four types based on MRI signs, and recommended surgical procedures according to the different type. However, the classification system only included the thoracic vertebrae. In 2008, a retrospective study analysed 76 patients with spinal tuberculosis, and put forward a new classification (GATA) according to abscesses, neurological deficit, vertebral collapse, kyphosis, spinal instability, and disc degeneration [5]. This new classification system is considered to be a practical guide for spinal tuberculosis treatment planning [26]. Nonetheless, the classification does not include spinal adnexal tuberculosis, as this criterion is too complex to master in the clinic. Non-surgical measures have been successful in the treatment of spinal tuberculosis patients without the use for bracing [16, 19].

Although surgical treatment for spinal tuberculosis abscess can lead to satisfactory clinical outcomes [27]. The treatment by CT puncture and catheterisation is minimally invasive, beneficial for the drainage of paravertebral abscesses, and reduce the possibility of conventional surgical debridement for tuberculosis abscesses. Moreover, this strategy reduces tuberculosis infection, consumption, pain, and complications related to prolonged bed rest, also enhanced recovery of mild spinal tuberculosis. Theoretically, it has advantages over single drug chemotherapy in the treatment of abscesses. The traditional anti-tuberculosis treatment or chemotherapy effect is always poor. For spinal tuberculosis accompanied by abscess, CT puncture intervention can not only drain abscess to reduce infection and consumption, pain and inflammation in the body at the early stage, but also facilitate the specimen of abscess taken out for further tuberculosis bacterial culture and drug resistance detection at the beginning of chemotherapy treatment, although it takes a long time to obtain the final result of drug sensitivity. The CT guided drainage of spinal tuberculosis accompanied by abscess could approach the individualization and reasonable chemotherapy regimen.

Timely diagnosis with clinical, imaging, microbiological, histopathological features and complete course of anti-tubercular treatment appears to be safe and effective for spinal tuberculosis [28]. At present, there is a lack of referential treatment method for screening drainage according to the definition of abscess volume. In the previous study, paravertebral abscess > 50 ml based on imaging was the important inclusion criteria. All patients achieved healing with no recurrence and no neurologic function impairment. ESR, VAS, kyphotic angle and oswestry disability index values decreased significantly. The CT guided drainage had been proven to be a safe and effective treatment for spinal tuberculosis with paravertebral abscesses [29]. In other anatomical regions, CT-guided percutaneous and drainage treatment was confirmed an efficient and attractive chose in the treatment of psoas abscesses to alternative the open surgical drainage, even multilocular pelvic and gluteal tuberculous abscesses [30, 31], which was an easy, safe and effective treatment of the thoracic and lumbar spinal tuberculosis with flow injection abscess, even in children [32, 33].

Although satisfactory outcomes were obtained in this study, several limitations exist. First, this was a retrospective study without random assignment of patients, a short follow-up period and small sample size, which may affect the reliability of the results. Second, the study did not include patients with neurological deficits, serious kyphosis, or large abscesses, which may cause a certain degree of bias. Third, need random controlled and prospective studies in future with large patient populations to prove how much volume of the paravertebral abscess was the best indication of CT-guided percutaneous drainage therapy for spinal tuberculosis.

## Conclusions

For patients diagnosed with mild spinal tuberculosis, conservative treatment can achieve satisfactory results. The strategy combined with CT-guided local chemotherapy treatment is minimally invasive, beneficial for the drainage of paravertebral abscesses, pain relief and enhanced recovery of mild spinal tuberculosis.

## Data Availability

All data used in the study are available at the request of the editors and reviewers. If someone wants to request the data from this study please contact M.D.Wenbo Diao.
